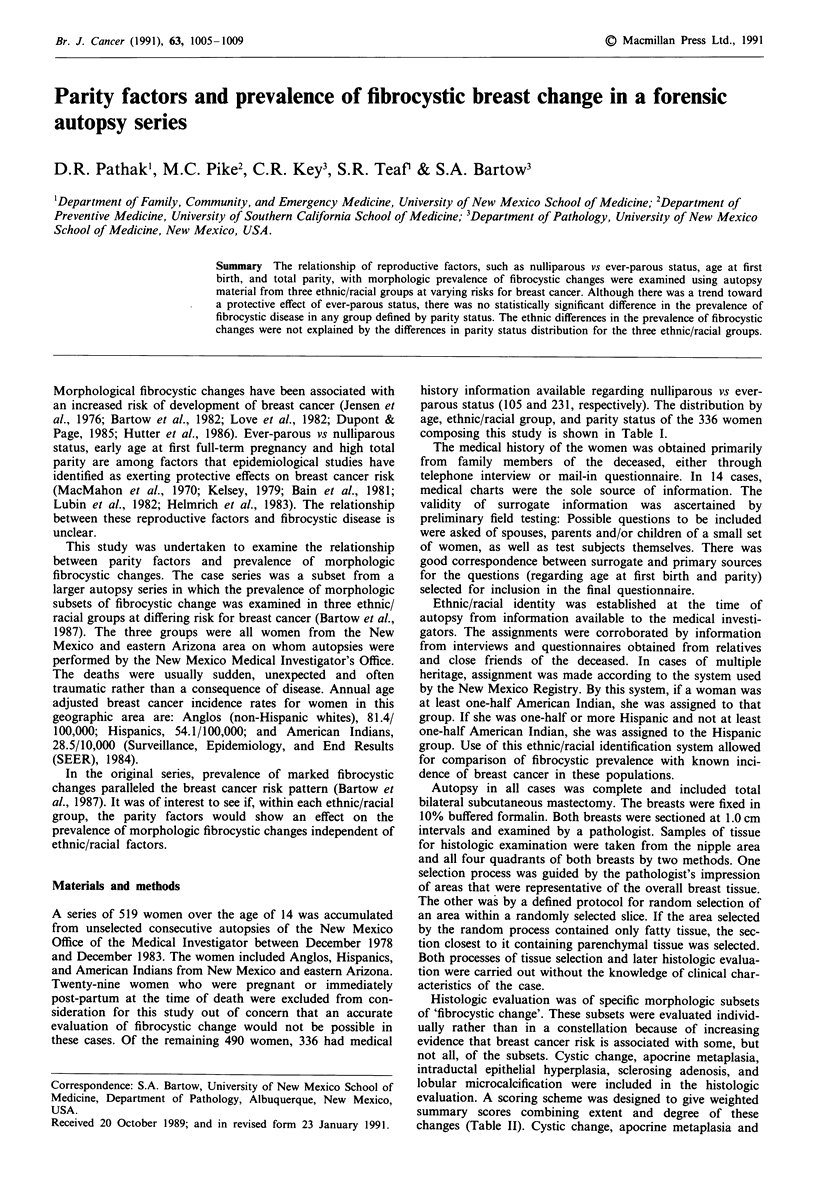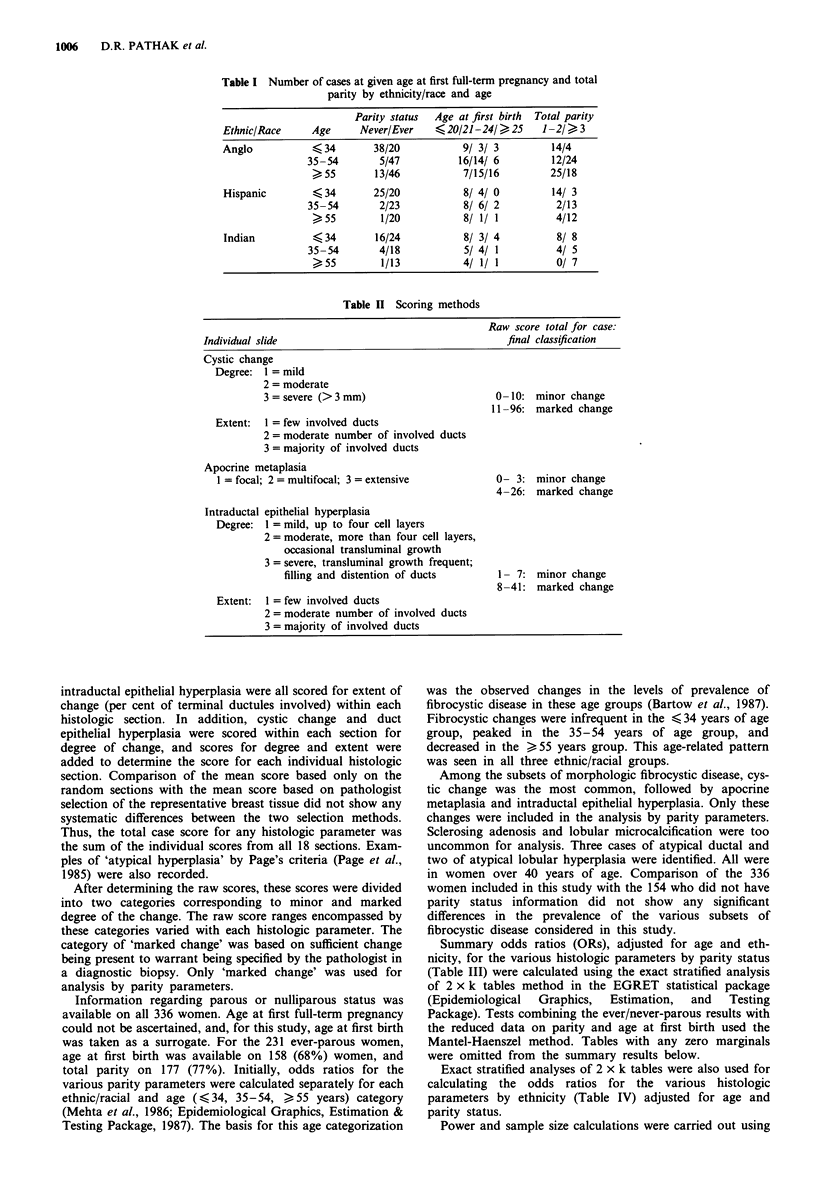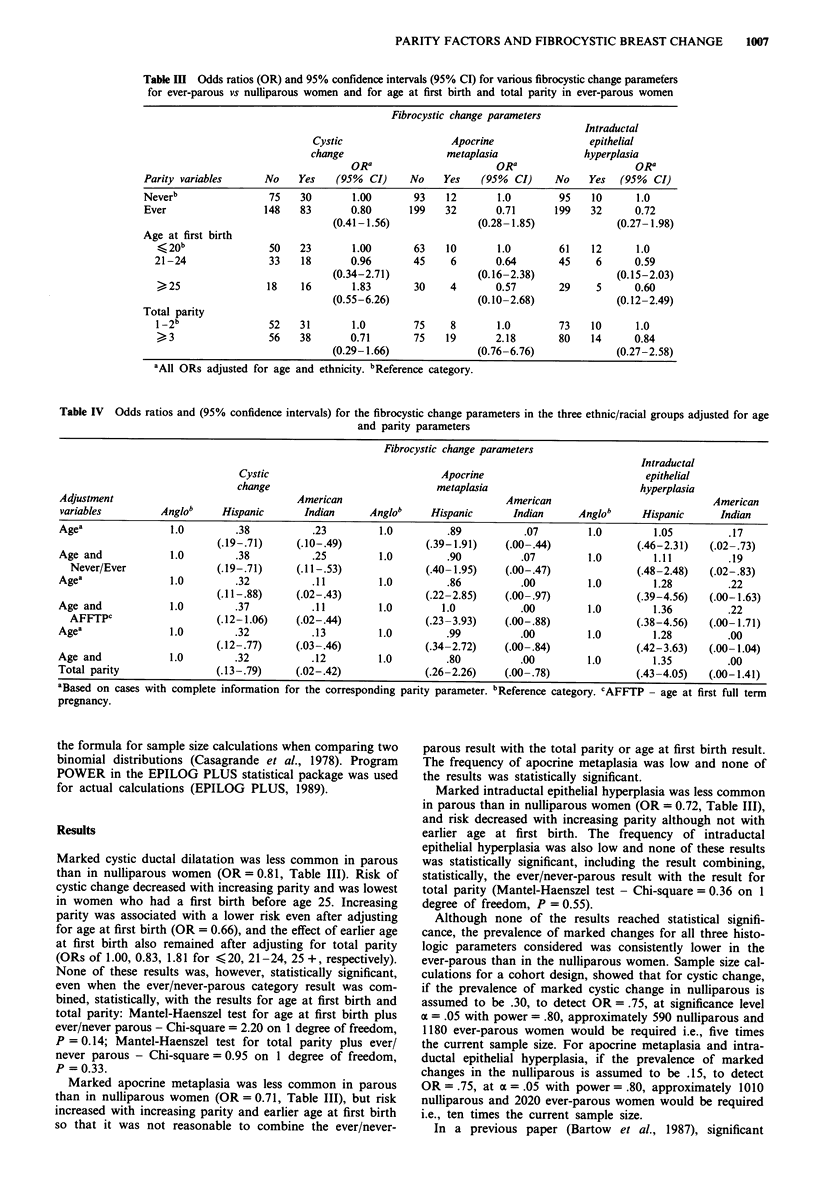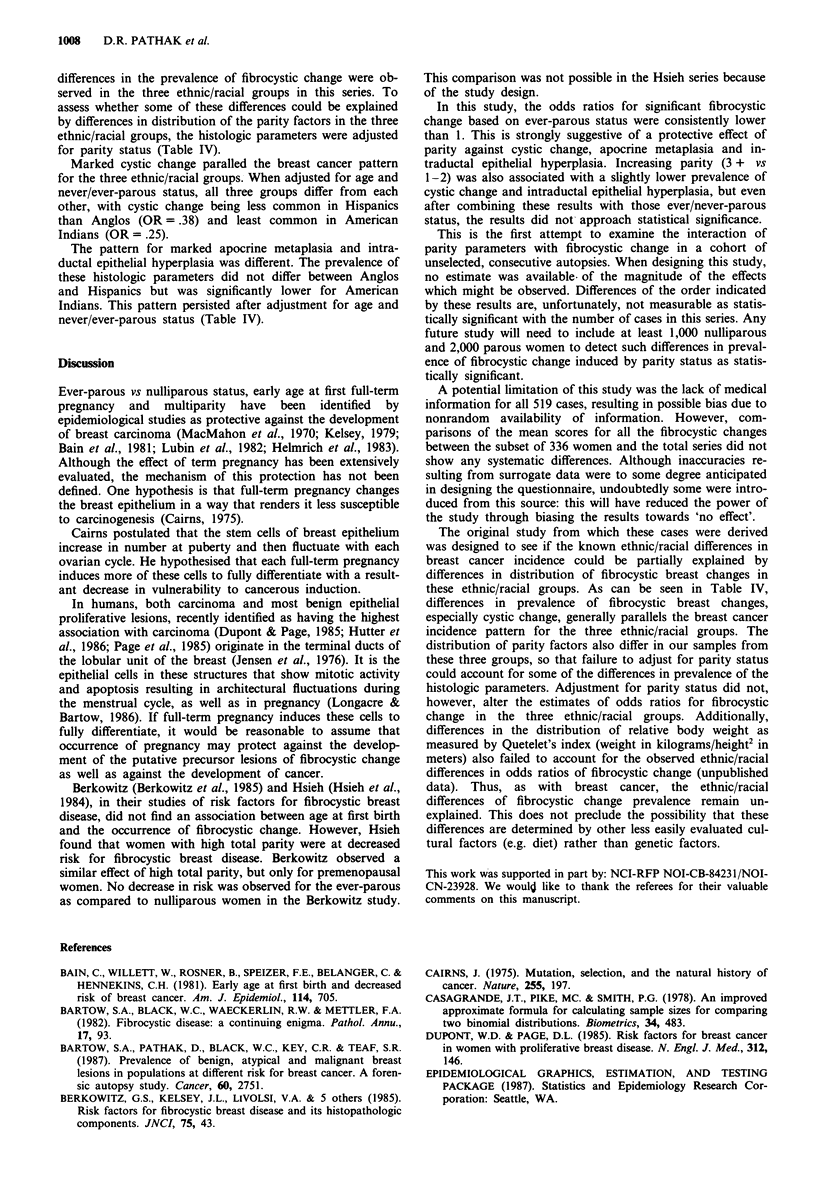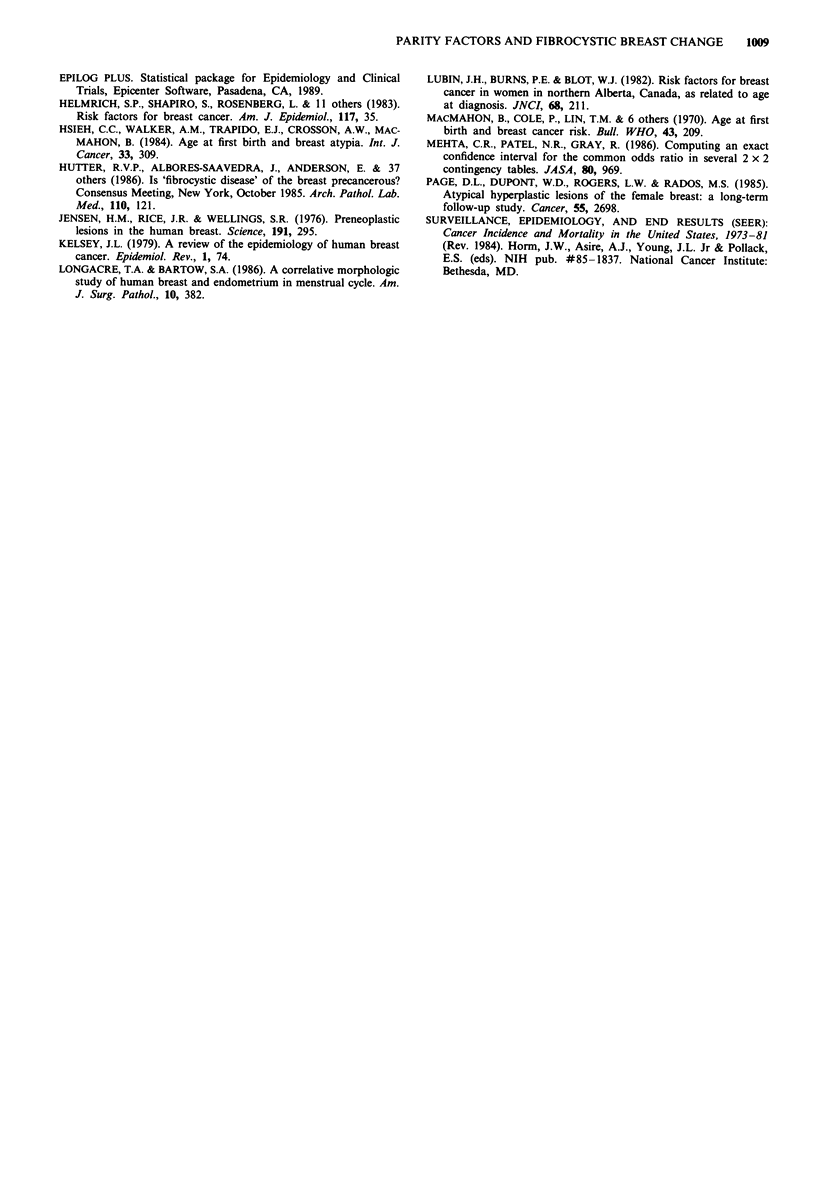# Parity factors and prevalence of fibrocystic breast change in a forensic autopsy series.

**DOI:** 10.1038/bjc.1991.218

**Published:** 1991-06

**Authors:** D. R. Pathak, M. C. Pike, C. R. Key, S. R. Teaf, S. A. Bartow

**Affiliations:** Department of Family, Community, University of New Mexico School of Medicine, Albuquerque.

## Abstract

The relationship of reproductive factors, such as nulliparous vs ever-parous status, age at first birth, and total parity, with morphologic prevalence of fibrocystic changes were examined using autopsy material from three ethnic/racial groups at varying risks for breast cancer. Although there was a trend toward a protective effect of ever-parous status, there was no statistically significant difference in the prevalence of fibrocystic disease in any group defined by parity status. The ethnic differences in the prevalence of fibrocystic changes were not explained by the differences in parity status distribution for the three ethnic/racial groups.


 © Macmillan Press Ltd., 1991

### Parity factors and prevalence of fibrocystic breast change in a forensic autopsy series

D.R. Pathak[1], M.C. Pike[2], C.R. Key[3], S.R. Teaf[1] & S.A. Bartow[3]

[1]*Department of Family, Community, and Emergency Medicine, University of New Mexico School of Medicine;* [2]*Department of Preventive Medicine, University of Southern California School of Medicine;* [3]*Department of Pathology, University of New Mexico School of Medicine, New Mexico, USA.*

**Summary** The relationship of reproductive factors, such as nulliparous *vs* ever-parous status, age at first birth, and total parity, with morphologic prevalence of fibrocystic changes were examined using autopsy material from three ethnic/racial groups at varying risks for breast cancer. Although there was a trend toward a protective effect of ever-parous status, there was no statistically significant difference in the prevalence of fibrocystic disease in any group defined by parity status. The ethnic differences in the prevalence of fibrocystic changes were not explained by the differences in parity status distribution for the three ethnic/racial groups.

Morphological fibrocystic changes have been associated with an increased risk of development of breast cancer (Jensen *et al.*, 1976; Bartow *et al.*, 1982; Love *et al.*, 1982; Dupont & Page, 1985; Hutter *et al.*, 1986). Ever-parous *vs* nulliparous status, early age at first full-term pregnancy and high total parity are among factors that epidemiological studies have identified as exerting protective effects on breast cancer risk (MacMahon *et al.*, 1970; Kelsey, 1979; Bain *et al.*, 1981; Lubin *et al.*, 1982; Helmrich *et al.*, 1983). The relationship between these reproductive factors and fibrocystic disease is unclear.

This study was undertaken to examine the relationship between parity factors and prevalence of morphologic fibrocystic changes. The case series was a subset from a larger autopsy series in which the prevalence of morphologic subsets of fibrocystic change was examined in three ethnic/racial groups at differing risk for breast cancer (Bartow *et al.*, 1987). The three groups were all women from the New Mexico and eastern Arizona area on whom autopsies were performed by the New Mexico Medical Investigator's Office. The deaths were usually sudden, unexpected and often traumatic rather than a consequence of disease. Annual age adjusted breast cancer incidence rates for women in this geographic area are: Anglos (non-Hispanic whites), 81.4/100,000; Hispanics, 54.1/100,000; and American Indians, 28.5/10,000 (Surveillance, Epidemiology, and End Results (SEER), 1984).

In the original series, prevalence of marked fibrocystic changes paralleled the breast cancer risk pattern (Bartow *et al.*, 1987). It was of interest to see if, within each ethnic/racial group, the parity factors would show an effect on the prevalence of morphologic fibrocystic changes independent of ethnic/racial factors.

### Materials and methods

A series of 519 women over the age of 14 was accumulated from unselected consecutive autopsies of the New Mexico Office of the Medical Investigator between December 1978 and December 1983. The women included Anglos, Hispanics, and American Indians from New Mexico and eastern Arizona. Twenty-nine women who were pregnant or immediately post-partum at the time of death were excluded from consideration for this study out of concern that an accurate evaluation of fibrocystic change would not be possible in these cases. Of the remaining 490 women, 336 had medical history information available regarding nulliparous *vs* ever-parous status (105 and 231, respectively). The distribution by age, ethnic/racial group, and parity status of the 336 women composing this study is shown in Table I.

The medical history of the women was obtained primarily from family members of the deceased, either through telephone interview or mail-in questionnaire. In 14 cases, medical charts were the sole source of information. The validity of surrogate information was ascertained by preliminary field testing: Possible questions to be included were asked of spouses, parents and/or children of a small set of women, as well as test subjects themselves. There was good correspondence between surrogate and primary sources for the questions (regarding age at first birth and parity) selected for inclusion in the final questionnaire.

Ethnic/racial identity was established at the time of autopsy from information available to the medical investigators. The assignments were corroborated by information from interviews and questionnaires obtained from relatives and close friends of the deceased. In cases of multiple heritage, assignment was made according to the system used by the New Mexico Registry. By this system, if a woman was at least one-half American Indian, she was assigned to that group. If she was one-half or more Hispanic and not at least one-half American Indian, she was assigned to the Hispanic group. Use of this ethnic/racial identification system allowed for comparison of fibrocystic prevalence with known incidence of breast cancer in these populations.

Autopsy in all cases was complete and included total bilateral subcutaneous mastectomy. The breasts were fixed in 10% buffered formalin. Both breasts were sectioned at 1.0 cm intervals and examined by a pathologist. Samples of tissue for histologic examination were taken from the nipple area and all four quadrants of both breasts by two methods. One selection process was guided by the pathologist's impression of areas that were representative of the overall breast tissue. The other was by a defined protocol for random selection of an area within a randomly selected slice. If the area selected by the random process contained only fatty tissue, the section closest to it containing parenchymal tissue was selected. Both processes of tissue selection and later histologic evaluation were carried out without the knowledge of clinical characteristics of the case.

Histologic evaluation was of specific morphologic subsets of 'fibrocystic change'. These subsets were evaluated individually rather than in a constellation because of increasing evidence that breast cancer risk is associated with some, but not all, of the subsets. Cystic change, apocrine metaplasia, intraductal epithelial hyperplasia, sclerosing adenosis, and lobular microcalcification were included in the histologic evaluation. A scoring scheme was designed to give weighted summary scores combining extent and degree of these changes (Table II). Cystic change, apocrine metaplasia and

Correspondence: S.A. Bartow, University of New Mexico School of Medicine, Department of Pathology, Albuquerque, New Mexico, USA.
Received 20 October 1989; and in revised form 23 January 1991.

**Table I** Number of cases at given age at first full-term pregnancy and total parity by ethnicity/race and age

| *Ethnic/Race* | *Age* | *Parity status Never/Ever* | *Age at first birth ≤20/21–24/≥25* | *Total parity 1–2/≥3* |
|---|---|---|---|---|
| Anglo | ≤34 | 38/20 | 9/ 3/ 3 | 14/4 |
| | 35–54 | 5/47 | 16/14/ 6 | 12/24 |
| | ≥55 | 13/46 | 7/15/16 | 25/18 |
| Hispanic | ≤34 | 25/20 | 8/ 4/ 0 | 14/ 3 |
| | 35–54 | 2/23 | 8/ 6/ 2 | 2/13 |
| | ≥55 | 1/20 | 8/ 1/ 1 | 4/12 |
| Indian | ≤34 | 16/24 | 8/ 3/ 4 | 8/ 8 |
| | 35–54 | 4/18 | 5/ 4/ 1 | 4/ 5 |
| | ≥55 | 1/13 | 4/ 1/ 1 | 0/ 7 |

**Table II** Scoring methods

| *Individual slide* | | *Raw score total for case: final classification* |
|---|---|---|
| Cystic change | | |
| Degree: | 1 = mild | |
| | 2 = moderate | |
| | 3 = severe (>3 mm) | 0–10: minor change |
| | | 11–96: marked change |
| Extent: | 1 = few involved ducts | |
| | 2 = moderate number of involved ducts | |
| | 3 = majority of involved ducts | |
| Apocrine metaplasia | | |
| | 1 = focal; 2 = multifocal; 3 = extensive | 0– 3: minor change |
| | | 4–26: marked change |
| Intraductal epithelial hyperplasia | | |
| Degree: | 1 = mild, up to four cell layers | |
| | 2 = moderate, more than four cell layers, occasional transluminal growth | |
| | 3 = severe, transluminal growth frequent; filling and distention of ducts | 1– 7: minor change |
| | | 8–41: marked change |
| Extent: | 1 = few involved ducts | |
| | 2 = moderate number of involved ducts | |
| | 3 = majority of involved ducts | |

intraductal epithelial hyperplasia were all scored for extent of change (per cent of terminal ductules involved) within each histologic section. In addition, cystic change and duct epithelial hyperplasia were scored within each section for degree of change, and scores for degree and extent were added to determine the score for each individual histologic section. Comparison of the mean score based only on the random sections with the mean score based on pathologist selection of the representative breast tissue did not show any systematic differences between the two selection methods. Thus, the total case score for any histologic parameter was the sum of the individual scores from all 18 sections. Examples of 'atypical hyperplasia' by Page's criteria (Page *et al.*, 1985) were also recorded.

After determining the raw scores, these scores were divided into two categories corresponding to minor and marked degree of the change. The raw score ranges encompassed by these categories varied with each histologic parameter. The category of 'marked change' was based on sufficient change being present to warrant being specified by the pathologist in a diagnostic biopsy. Only 'marked change' was used for analysis by parity parameters.

Information regarding parous or nulliparous status was available on all 336 women. Age at first full-term pregnancy could not be ascertained, and, for this study, age at first birth was taken as a surrogate. For the 231 ever-parous women, age at first birth was available on 158 (68%) women, and total parity on 177 (77%). Initially, odds ratios for the various parity parameters were calculated separately for each ethnic/racial and age (≤34, 35–54, ≥55 years) category (Mehta *et al.*, 1986; Epidemiological Graphics, Estimation & Testing Package, 1987). The basis for this age categorization was the observed changes in the levels of prevalence of fibrocystic disease in these age groups (Bartow *et al.*, 1987). Fibrocystic changes were infrequent in the ≤34 years of age group, peaked in the 35–54 years of age group, and decreased in the ≥55 years group. This age-related pattern was seen in all three ethnic/racial groups.

Among the subsets of morphologic fibrocystic disease, cystic change was the most common, followed by apocrine metaplasia and intraductal epithelial hyperplasia. Only these changes were included in the analysis by parity parameters. Sclerosing adenosis and lobular microcalcification were too uncommon for analysis. Three cases of atypical ductal and two of atypical lobular hyperplasia were identified. All were in women over 40 years of age. Comparison of the 336 women included in this study with the 154 who did not have parity status information did not show any significant differences in the prevalence of the various subsets of fibrocystic disease considered in this study.

Summary odds ratios (ORs), adjusted for age and ethnicity, for the various histologic parameters by parity status (Table III) were calculated using the exact stratified analysis of 2 × k tables method in the EGRET statistical package (Epidemiological Graphics, Estimation, and Testing Package). Tests combining the ever/never-parous results with the reduced data on parity and age at first birth used the Mantel-Haenszel method. Tables with any zero marginals were omitted from the summary results below.

Exact stratified analyses of 2 × k tables were also used for calculating the odds ratios for the various histologic parameters by ethnicity (Table IV) adjusted for age and parity status.

Power and sample size calculations were carried out using

**Table III** Odds ratios (OR) and 95% confidence intervals (95% CI) for various fibrocystic change parameters for ever-parous *vs* nulliparous women and for age at first birth and total parity in ever-parous women

| | *Fibrocystic change parameters* | | | | | | | | |
|---|---|---|---|---|---|---|---|---|---|
| | *Cystic change* | | | *Apocrine metaplasia* | | | *Intraductal epithelial hyperplasia* | | |
| *Parity variables* | *No* | *Yes* | *OR[a] (95% CI)* | *No* | *Yes* | *OR[a] (95% CI)* | *No* | *Yes* | *OR[a] (95% CI)* |
| Never[b] | 75 | 30 | 1.00 | 93 | 12 | 1.0 | 95 | 10 | 1.0 |
| Ever | 148 | 83 | 0.80 (0.41–1.56) | 199 | 32 | 0.71 (0.28–1.85) | 199 | 32 | 0.72 (0.27–1.98) |
| Age at first birth | | | | | | | | | |
| ⩽20[b] | 50 | 23 | 1.00 | 63 | 10 | 1.0 | 61 | 12 | 1.0 |
| 21–24 | 33 | 18 | 0.96 (0.34–2.71) | 45 | 6 | 0.64 (0.16–2.38) | 45 | 6 | 0.59 (0.15–2.03) |
| ⩾25 | 18 | 16 | 1.83 (0.55–6.26) | 30 | 4 | 0.57 (0.10–2.68) | 29 | 5 | 0.60 (0.12–2.49) |
| Total parity | | | | | | | | | |
| 1–2[b] | 52 | 31 | 1.0 | 75 | 8 | 1.0 | 73 | 10 | 1.0 |
| ⩾3 | 56 | 38 | 0.71 (0.29–1.66) | 75 | 19 | 2.18 (0.76–6.76) | 80 | 14 | 0.84 (0.27–2.58) |

[a]All ORs adjusted for age and ethnicity. [b]Reference category.

**Table IV** Odds ratios and (95% confidence intervals) for the fibrocystic change parameters in the three ethnic/racial groups adjusted for age and parity parameters

| | *Fibrocystic change parameters* | | | | | | | | |
|---|---|---|---|---|---|---|---|---|---|
| | *Cystic change* | | | *Apocrine metaplasia* | | | *Intraductal epithelial hyperplasia* | | |
| *Adjustment variables* | *Anglo[b]* | *Hispanic* | *American Indian* | *Anglo[b]* | *Hispanic* | *American Indian* | *Anglo[b]* | *Hispanic* | *American Indian* |
| Age[a] | 1.0 | .38 (.19–.71) | .23 (.10–.49) | 1.0 | .89 (.39–1.91) | .07 (.00–.44) | 1.0 | 1.05 (.46–2.31) | .17 (.02–.73) |
| Age and Never/Ever | 1.0 | .38 (.19–.71) | .25 (.11–.53) | 1.0 | .90 (.40–1.95) | .07 (.00–.47) | 1.0 | 1.11 (.48–2.48) | .19 (.02–.83) |
| Age[a] | 1.0 | .32 (.11–.88) | .11 (.02–.43) | 1.0 | .86 (.22–2.85) | .00 (.00–.97) | 1.0 | 1.28 (.39–4.56) | .22 (.00–1.63) |
| Age and AFFTP[c] | 1.0 | .37 (.12–1.06) | .11 (.02–.44) | 1.0 | 1.0 (.23–3.93) | .00 (.00–.88) | 1.0 | 1.36 (.38–4.56) | .22 (.00–1.71) |
| Age[a] | 1.0 | .32 (.12–.77) | .13 (.03–.46) | 1.0 | .99 (.34–2.72) | .00 (.00–.84) | 1.0 | 1.28 (.42–3.63) | .00 (.00–1.04) |
| Age and Total parity | 1.0 | .32 (.13–.79) | .12 (.02–.42) | 1.0 | .80 (.26–2.26) | .00 (.00–.78) | 1.0 | 1.35 (.43–4.05) | .00 (.00–1.41) |

[a]Based on cases with complete information for the corresponding parity parameter. [b]Reference category. [c]AFFTP – age at first full term pregnancy.

the formula for sample size calculations when comparing two binomial distributions (Casagrande *et al.*, 1978). Program POWER in the EPILOG PLUS statistical package was used for actual calculations (EPILOG PLUS, 1989).

### Results

Marked cystic ductal dilatation was less common in parous than in nulliparous women (OR = 0.81, Table III). Risk of cystic change decreased with increasing parity and was lowest in women who had a first birth before age 25. Increasing parity was associated with a lower risk even after adjusting for age at first birth (OR = 0.66), and the effect of earlier age at first birth also remained after adjusting for total parity (ORs of 1.00, 0.83, 1.81 for ⩽20, 21–24, 25 +, respectively). None of these results was, however, statistically significant, even when the ever/never-parous category result was combined, statistically, with the results for age at first birth and total parity: Mantel-Haenszel test for age at first birth plus ever/never parous – Chi-square = 2.20 on 1 degree of freedom, $P = 0.14$; Mantel-Haenszel test for total parity plus ever/never parous – Chi-square = 0.95 on 1 degree of freedom, $P = 0.33$.

Marked apocrine metaplasia was less common in parous than in nulliparous women (OR = 0.71, Table III), but risk increased with increasing parity and earlier age at first birth so that it was not reasonable to combine the ever/never-parous result with the total parity or age at first birth result. The frequency of apocrine metaplasia was low and none of the results was statistically significant.

Marked intraductal epithelial hyperplasia was less common in parous than in nulliparous women (OR = 0.72, Table III), and risk decreased with increasing parity although not with earlier age at first birth. The frequency of intraductal epithelial hyperplasia was also low and none of these results was statistically significant, including the result combining, statistically, the ever/never-parous result with the result for total parity (Mantel-Haenszel test – Chi-square = 0.36 on 1 degree of freedom, $P = 0.55$).

Although none of the results reached statistical significance, the prevalence of marked changes for all three histologic parameters considered was consistently lower in the ever-parous than in the nulliparous women. Sample size calculations for a cohort design, showed that for cystic change, if the prevalence of marked cystic change in nulliparous is assumed to be .30, to detect OR = .75, at significance level $\alpha = .05$ with power = .80, approximately 590 nulliparous and 1180 ever-parous women would be required i.e., five times the current sample size. For apocrine metaplasia and intraductal epithelial hyperplasia, if the prevalence of marked changes in the nulliparous is assumed to be .15, to detect OR = .75, at $\alpha = .05$ with power = .80, approximately 1010 nulliparous and 2020 ever-parous women would be required i.e., ten times the current sample size.

In a previous paper (Bartow *et al.*, 1987), significant differences in the prevalence of fibrocystic change were observed in the three ethnic/racial groups in this series. To assess whether some of these differences could be explained by differences in distribution of the parity factors in the three ethnic/racial groups, the histologic parameters were adjusted for parity status (Table IV).

Marked cystic change paralled the breast cancer pattern for the three ethnic/racial groups. When adjusted for age and never/ever-parous status, all three groups differ from each other, with cystic change being less common in Hispanics than Anglos (OR = .38) and least common in American Indians (OR = .25).

The pattern for marked apocrine metaplasia and intraductal epithelial hyperplasia was different. The prevalence of these histologic parameters did not differ between Anglos and Hispanics but was significantly lower for American Indians. This pattern persisted after adjustment for age and never/ever-parous status (Table IV).

### Discussion

Ever-parous *vs* nulliparous status, early age at first full-term pregnancy and multiparity have been identified by epidemiological studies as protective against the development of breast carcinoma (MacMahon *et al.*, 1970; Kelsey, 1979; Bain *et al.*, 1981; Lubin *et al.*, 1982; Helmrich *et al.*, 1983). Although the effect of term pregnancy has been extensively evaluated, the mechanism of this protection has not been defined. One hypothesis is that full-term pregnancy changes the breast epithelium in a way that renders it less susceptible to carcinogenesis (Cairns, 1975).

Cairns postulated that the stem cells of breast epithelium increase in number at puberty and then fluctuate with each ovarian cycle. He hypothesised that each full-term pregnancy induces more of these cells to fully differentiate with a resultant decrease in vulnerability to cancerous induction.

In humans, both carcinoma and most benign epithelial proliferative lesions, recently identified as having the highest association with carcinoma (Dupont & Page, 1985; Hutter *et al.*, 1986; Page *et al.*, 1985) originate in the terminal ducts of the lobular unit of the breast (Jensen *et al.*, 1976). It is the epithelial cells in these structures that show mitotic activity and apoptosis resulting in architectural fluctuations during the menstrual cycle, as well as in pregnancy (Longacre & Bartow, 1986). If full-term pregnancy induces these cells to fully differentiate, it would be reasonable to assume that occurrence of pregnancy may protect against the development of the putative precursor lesions of fibrocystic change as well as against the development of cancer.

Berkowitz (Berkowitz *et al.*, 1985) and Hsieh (Hsieh *et al.*, 1984), in their studies of risk factors for fibrocystic breast disease, did not find an association between age at first birth and the occurrence of fibrocystic change. However, Hsieh found that women with high total parity were at decreased risk for fibrocystic breast disease. Berkowitz observed a similar effect of high total parity, but only for premenopausal women. No decrease in risk was observed for the ever-parous as compared to nulliparous women in the Berkowitz study. This comparison was not possible in the Hsieh series because of the study design.

In this study, the odds ratios for significant fibrocystic change based on ever-parous status were consistently lower than 1. This is strongly suggestive of a protective effect of parity against cystic change, apocrine metaplasia and intraductal epithelial hyperplasia. Increasing parity (3+ *vs* 1–2) was also associated with a slightly lower prevalence of cystic change and intraductal epithelial hyperplasia, but even after combining these results with those ever/never-parous status, the results did not approach statistical significance.

This is the first attempt to examine the interaction of parity parameters with fibrocystic change in a cohort of unselected, consecutive autopsies. When designing this study, no estimate was available of the magnitude of the effects which might be observed. Differences of the order indicated by these results are, unfortunately, not measurable as statistically significant with the number of cases in this series. Any future study will need to include at least 1,000 nulliparous and 2,000 parous women to detect such differences in prevalence of fibrocystic change induced by parity status as statistically significant.

A potential limitation of this study was the lack of medical information for all 519 cases, resulting in possible bias due to nonrandom availability of information. However, comparisons of the mean scores for all the fibrocystic changes between the subset of 336 women and the total series did not show any systematic differences. Although inaccuracies resulting from surrogate data were to some degree anticipated in designing the questionnaire, undoubtedly some were introduced from this source: this will have reduced the power of the study through biasing the results towards 'no effect'.

The original study from which these cases were derived was designed to see if the known ethnic/racial differences in breast cancer incidence could be partially explained by differences in distribution of fibrocystic breast changes in these ethnic/racial groups. As can be seen in Table IV, differences in prevalence of fibrocystic breast changes, especially cystic change, generally parallels the breast cancer incidence pattern for the three ethnic/racial groups. The distribution of parity factors also differ in our samples from these three groups, so that failure to adjust for parity status could account for some of the differences in prevalence of the histologic parameters. Adjustment for parity status did not, however, alter the estimates of odds ratios for fibrocystic change in the three ethnic/racial groups. Additionally, differences in the distribution of relative body weight as measured by Quetelet's index (weight in kilograms/height$^2$ in meters) also failed to account for the observed ethnic/racial differences in odds ratios of fibrocystic change (unpublished data). Thus, as with breast cancer, the ethnic/racial differences of fibrocystic change prevalence remain unexplained. This does not preclude the possibility that these differences are determined by other less easily evaluated cultural factors (e.g. diet) rather than genetic factors.

This work was supported in part by: NCI-RFP NOI-CB-84231/NOI-CN-23928. We would like to thank the referees for their valuable comments on this manuscript.

### References

BAIN, C., WILLETT, W., ROSNER, B., SPEIZER, F.E., BELANGER, C. & HENNEKINS, C.H. (1981). Early age at first birth and decreased risk of breast cancer. *Am. J. Epidemiol.*, **114**, 705.

BARTOW, S.A., BLACK, W.C., WAECKERLIN, R.W. & METTLER, F.A. (1982). Fibrocystic disease: a continuing enigma. *Pathol. Annu.*, **17**, 93.

BARTOW, S.A., PATHAK, D., BLACK, W.C., KEY, C.R. & TEAF, S.R. (1987). Prevalence of benign, atypical and malignant breast lesions in populations at different risk for breast cancer. A forensic autopsy study. *Cancer*, **60**, 2751.

BERKOWITZ, G.S., KELSEY, J.L., LIVOLSI, V.A. & 5 others (1985). Risk factors for fibrocystic breast disease and its histopathologic components. *JNCI*, **75**, 43.

CAIRNS, J. (1975). Mutation, selection, and the natural history of cancer. *Nature*, **255**, 197.

CASAGRANDE, J.T., PIKE, MC. & SMITH, P.G. (1978). An improved approximate formula for calculating sample sizes for comparing two binomial distributions. *Biometrics*, **34**, 483.

DUPONT, W.D. & PAGE, D.L. (1985). Risk factors for breast cancer in women with proliferative breast disease. *N. Engl. J. Med.*, **312**, 146.

EPIDEMIOLOGICAL GRAPHICS, ESTIMATION, AND TESTING PACKAGE (1987). Statistics and Epidemiology Research Corporation: Seattle, WA.

EPILOG PLUS. Statistical package for Epidemiology and Clinical Trials, Epicenter Software, Pasadena, CA, 1989.

HELMRICH, S.P., SHAPIRO, S., ROSENBERG, L. & 11 others (1983). Risk factors for breast cancer. *Am. J. Epidemiol.*, **117**, 35.

HSIEH, C.C., WALKER, A.M., TRAPIDO, E.J., CROSSON, A.W., MACMAHON, B. (1984). Age at first birth and breast atypia. *Int. J. Cancer*, **33**, 309.

HUTTER, R.V.P., ALBORES-SAAVEDRA, J., ANDERSON, E. & 37 others (1986). Is 'fibrocystic disease' of the breast precancerous? Consensus Meeting, New York, October 1985. *Arch. Pathol. Lab. Med.*, **110**, 121.

JENSEN, H.M., RICE, J.R. & WELLINGS, S.R. (1976). Preneoplastic lesions in the human breast. *Science*, **191**, 295.

KELSEY, J.L. (1979). A review of the epidemiology of human breast cancer. *Epidemiol. Rev.*, **1**, 74.

LONGACRE, T.A. & BARTOW, S.A. (1986). A correlative morphologic study of human breast and endometrium in menstrual cycle. *Am. J. Surg. Pathol.*, **10**, 382.

LUBIN, J.H., BURNS, P.E. & BLOT, W.J. (1982). Risk factors for breast cancer in women in northern Alberta, Canada, as related to age at diagnosis. *JNCI*, **68**, 211.

MACMAHON, B., COLE, P., LIN, T.M. & 6 others (1970). Age at first birth and breast cancer risk. *Bull. WHO*, **43**, 209.

MEHTA, C.R., PATEL, N.R., GRAY, R. (1986). Computing an exact confidence interval for the common odds ratio in several 2 × 2 contingency tables. *JASA*, **80**, 969.

PAGE, D.L., DUPONT, W.D., ROGERS, L.W. & RADOS, M.S. (1985). Atypical hyperplastic lesions of the female breast: a long-term follow-up study. *Cancer*, **55**, 2698.

SURVEILLANCE, EPIDEMIOLOGY, AND END RESULTS (SEER): *Cancer Incidence and Mortality in the United States, 1973–81* (Rev. 1984). Horm, J.W., Asire, A.J., Young, J.L. Jr & Pollack, E.S. (eds). NIH pub. #85-1837. National Cancer Institute: Bethesda, MD.